# Contaminated Heparin and Outcomes after Cardiac Surgery: A Retrospective Propensity-Matched Cohort Study

**DOI:** 10.1371/journal.pone.0106096

**Published:** 2014-08-27

**Authors:** Heiko A. Kaiser, Arbi Ben Abdallah, Nan Lin, Bethany R. Tellor, Mohammad Helwani, Jennifer R. Smith, Marc R. Moon, Michael S. Avidan

**Affiliations:** 1 Department of Anesthesiology, Washington University in Saint Louis School of Medicine, Saint Louis, Missouri, United States of America; 2 Department of Mathematics, Washington University in Saint Louis, Saint Louis, Missouri, United States of America; 3 Department of Pharmacy, Barnes-Jewish Hospital, Saint Louis, Missouri, United States of America; 4 Department of Cardiothoracic Surgery, Washington University in Saint Louis School of Medicine, Saint Louis, Missouri, United States of America; San Raffaele Scientific Institute, Italy

## Abstract

**Background:**

During 2007 and 2008 it is likely that millions of patients in the US received heparin contaminated (CH) with oversulfated chondroitin sulfate, which was associated with anaphylactoid reactions. We tested the hypothesis that CH was associated with serious morbidity, mortality, intensive care unit (ICU) stay and heparin-induced thrombocytopenia following adult cardiac surgery.

**Methods and Findings:**

We conducted a single center, retrospective, propensity-matched cohort study during the period of CH and the equivalent time frame in the three preceding or the two following years. Perioperative data were obtained from the institutional record of the Society of Thoracic Surgeons National Database, for which the data collection is prospective, standardized and performed by independent investigators. After matching, logistic regression was performed to evaluate the independent effect of CH on the composite adverse outcome (myocardial infarction, stroke, pneumonia, dialysis, cardiac arrest) and on mortality. Cox regression was used to determine the association between CH and ICU length of stay. The 1∶5 matched groups included 220 patients potentially exposed to CH and 918 controls. There were more adverse outcomes in the exposed cohort (20.9% versus 12.0%; difference = 8.9%; 95% CI 3.6% to 15.1%, P<0.001) with an odds ratio for CH of 2.0 (95% CI, 1.4 to 3.0, P<0.001). In the exposed group there was a non-significant increase in mortality (5.9% versus 3.5%, difference = 2.4%; 95% CI, −0.4 to 3.5%, P = 0.1), the median ICU stay was longer by 14.1 hours (interquartile range −26.6 to 79.8, S = 3299, P = 0.0004) with an estimated hazard ratio for CH of 1.2 (95% CI, 1.0 to 1.4, P = 0.04). There was no difference in nadir platelet counts between cohorts.

**Conclusions:**

The results from this single center study suggest the possibility that contaminated heparin might have contributed to serious morbidity following cardiac surgery.

## Introduction

In March 2008, the Federal Drug Administration (FDA) announced that heparin from one of the major manufacturers had been contaminated with oversulfated chondroitin sulfate (OSCS). Suspicion for contamination arose because several anaphylactoid reactions to heparin were reported, whereas heparin, one of the most commonly administered hospital medications, is rarely associated with such reactions. The anaphylactoid reactions were most likely to have been caused by activation of the kinin-kallikrein system leading to production of the vasodilator bradykinin, and to stimulation of the alternative complement pathway leading to synthesis of the potent anaphylotoxins C3a and C5a [1]–[3].

When contamination was suspected, the manufacturer withdrew its heparin. However, during the period that OSCS contaminated heparin was in circulation, the FDA estimated that up to 50% of the heparin in the US was from the manufacturer of contaminated heparin [2]. Patients undergoing cardiac surgical procedures typically receive large bolus doses (300 to 400 units/kg) of heparin in order to achieve intense anticoagulation for cardiopulmonary bypass. It is probable that many of the most profound anaphylactoid reactions would have occurred in this patient population. It is currently unknown specifically whether patient outcomes from cardiac surgery were worse during the period of OSCS contamination. Also it is generally unknown whether appropriately managed anaphylactoid reactions are associated with increased morbidity and mortality beyond the acute episode. OSCS contaminated heparin has been hypothesized to increase the incidence of heparin-induced thrombocytopenia [4], [5], which occurs relatively frequently in cardiac surgery patients [6]. Interestingly, exogenous bradykinin might have a cardioprotective role in cardiac surgery by attenuating myocardial injury and through anti-inflammatory actions [7]. It is thus also important to consider that OSCS heparin, through production of bradykinin, might have improved outcomes after cardiac surgery.

We therefore conducted a single center propensity-matched study including adult cardiac surgery patients to determine whether during the period of OSCS heparin contamination there were differences in morbidity, mortality, intensive care unit stay (ICU), or heparin-induced thrombocytopenia following cardiac surgery.

## Methods

### Study Design

This was a retrospective cohort study involving adult patients undergoing cardiac surgery at Barnes-Jewish Hospital in St. Louis, Missouri. The Washington University Human Research Protection Office approved the study and waived the requirement for informed consent. The STROBE checklist for observational studies was used to guide the methods of this study and to structure this manuscript [8].

The investigation was designed to compare surgical patients during the period of heparin contamination (potentially exposed group) to patients who had their surgery during the equivalent time frame in the three preceding or the two subsequent years (control group). The time period inclusion criterion was to limit the possibility of seasonal disparities in patient outcomes biasing the results [9]. The first reports about serious adverse events or deaths potentially caused by OSCS were submitted in November 2007, and the manufacturer recalled 9 lots of multi-dose heparin vials on January 17th, 2008. The remaining multi- and single-dose vials of heparin were recalled on February 28th, 2008. Based on pharmacy invoices for this time period, all multi-dose vials of heparin received by Barnes-Jewish Hospital were on the recall list. Typically, heparin for cardiac surgery is administered from these multi-dose vials, but the exact lot number of heparin for each patient is not recorded. Therefore adult cardiac surgery patients at Barnes-Jewish Hospital potentially received contaminated heparin from November 2007 to February 2008.

Perioperative data were obtained from the institutional record of the Society of Thoracic Surgeons National Database, for which the data collection is prospective, standardized and performed by independent investigators [10]. Patients older than eighteen undergoing open cardiac surgical procedures requiring heparin anticoagulation between November 1st and February 28th during the years 2004 to 2010 were included. Patients with missing information regarding demographics or perioperative variables that were used for matching or regression analysis were excluded.

The primary outcome was a composite postoperative morbidity outcome including myocardial infarction, stroke, pneumonia, renal failure, and cardiac arrest during each patient’s hospital stay as well as in-hospital mortality. As secondary outcomes we analyzed Intensive Care Unit length of stay and the lowest recorded platelet count during the first ten postoperative days to compare severity of thrombocytopenia. Except for platelet count these outcomes are defined in the Society of Thoracic Surgery database [11]. A subgroup analysis for patients taking angiotensin-converting-enzyme-inhibitors or angiotensin-receptor-blocker was planned, as the angiotensin-converting-enzyme-inhibitors increase bradykinin concentrations and could therefore modify the effects of OSCS contaminated heparin [12]. But there is no distinction between these medications in the Society of Thoracic Surgery database.

### Statistical Analysis

The differences in patient characteristics, co-morbidities and details of the scheduled surgery between the potentially exposed and the control group were evaluated with a student’s t-test and chi-square test as deemed appropriate. Normality of continuous variables was verified with one-sided Kolmogorov-Smirnov tests before parametric statistics were applied. To further assess the potential imbalance between the cohorts, the standardized differences in means for each of the 24 variables was calculated (Table S1). Due to the imbalances, propensity score matching was done with up to five control patients for each potentially exposed case based on characteristics, co-morbid covariates and surgical details (Table S1). The nearest neighbor matching without replacement was used with a caliper of 0.2 standard deviations of the logit of a patient’s predicted propensity score, because it resulted in the overall best balance and the smallest reduction in number of potentially exposed patients. To account for the fact that some potentially exposed cases could just be matched to one control, weights were generated to correct for this disproportion. Every potentially exposed patient received a weight of one and the weights for the matched control units were calculated by n_exposedi_/n_controli_, where n_exposedi_ and n_controli_ were the number of potentially exposed and control units in stratum i. The control group weights were scaled then to sum to the number of uniquely matched control units. Following propensity score matching the balance of the matched potentially exposed and control groups was evaluated by calculating variance ratios, absolute values of the standardized differences in means as well as Hotelling’s test. Furthermore, a weighted t-tests or chi-square test were performed as appropriate [13]–[17].

Unadjusted analyses were initially conducted for all outcomes. To depict the influence of the propensity score matching on the differences in outcomes, a chi-square or Wilcoxon Rank-Sum test was used before, and a weighted McNemar’s test or Wilcoxon Signed Rank test after matching as indicated. For the subgroup analyses of angiotensin-converting-enzyme inhibitor or angiotensin-receptor blocker versus events of composite outcome a weighted chi-square test was applied.

Adjusted analyses comprised conditional multivariable logistic regression using data from the matched patient cohort to evaluate the independent effect of contaminated heparin on the composite outcome and on mortality. Proportional hazards Cox regression was used to determine the association between contaminated heparin and ICU length of stay. We checked proportionality of hazards by testing for non-zero slopes in a generalized linear regression of the scaled Schoenfeld residuals on functions of time, and additionally graphically by eye. Covariates were defined a-priori by their clinical significance in the Euroscore II [18]. The number of covariates per logistic regression model was restricted to at least 10 events per covariate to decrease the likelihood of overfitting. Covariates included heparin – uncontaminated or potentially contaminated, age (categorical with 3 levels), race (dichotomous), number of co-morbidities (categorical with 3 levels), left ventricular ejection fraction (categorical with 3 levels), cardiopulmonary bypass time (categorical with 3 levels), Tranexamic acid (dichotomous), and surgeon experience (dichotomous: Junior (instructor or assistant professor) versus senior (associate or full professor) surgeon). Only heparin, co-morbidities, left ventricular ejection fraction, and cardiopulmonary bypass time were used as covariates for the mortality logistic regression model. All variables were entered with the main factor of interest, heparin exposure, in a single step of the model. Goodness of fit for the logistic regression was assessed with the log likelihood ratio, Wald and Hosmer-Lemeshow tests. For the proportional hazards Cox regression the proportionality assumption was checked. A p-value <0.05 was considered significant in all applied tests. SPSS Version 21 (IBM, Armonk, New York) was used for data handling, and R (The R Foundation for Statistical Computing, Version 2.15.3) with the packages MatchIt [16] and Survey [19] was used for statistical analyses. At the request of the handling editor, using the raw data, the statistical analyses were repeated by an independent statistician (NL). The approaches used for these independent analyses were similar to those used in the initial analyses.

## Results

Of the 1417 patients meeting the inclusion criteria there were 220 during the period of contaminated heparin (2007 to 2008) and 1197 during the corresponding period in the three preceding and two subsequent years. Seven patients were excluded from the initial database (n = 1424) due to missing covariate information (0.49%). The two groups differed significantly in certain characteristics and co-morbidities (Table S1). This resulted in a significantly different overall propensity score of 0.15 in the control group and 0.19 in the potentially exposed group (P<0.001). Following propensity score matching there were no significant differences between the groups with standardized mean difference not exceeding 0.25, no variance ratio outside the range of 0.5–2, and a non-significant Hotelling’s test (P = 0.985). Of the control patients, 279 were not suitable for matching. Interestingly, correction of the imbalances through matching did not substantially alter the findings for the composite postoperative complication outcome (Table 1).

**Table 1:** Summary Statistics of Unadjusted Patient Outcome Before and After PS Matching during Heparin Contamination.

	Before PS Matching			After PS Matching		
	Control	Exposed		Control	Exposed	
	(n = 1197)	(n = 220)		(n = 918)	(n = 220)	
Outcome	n(%) or M(IQR)	n(%) or M(IQR)	P	n(%) or M(IQR)	n(%) or M(IQR)	P
Composite Outcome*	172 (14.4%)	46 (20.9%)	0.01	110 (12.0%)	46 (20.9%)	<0.001
*- MI*	8 (0.7%)	4 (1.8%)	0.09	8 (0.9%)	4 (1.8%)	0.20
*- Stroke*	33 (2.8%)	9 (4.1%)	0.28	20 (2.2%)	9 (4.1%)	0.11
*- Pneumonia*	120 (10.0%)	33 (15.0%)	0.03	76 (8.3%)	33 (15.0%)	0.003
*- Dialysis*	33 (2.8%)	8 (3.6%)	0.47	23 (2.5%)	8 (3.6%)	0.35
*- Cardiac Arrest*	27 (2.3%)	6 (2.7%)	0.67	17 (1.9%)	6 (2.7%)	0.43
In-Hospital Mortality	51 (4.3%)	13 (5.9%)	0.29	32 (3.5%)	13 (5.9%)	0.10
ICU-LOS in hours	55.0 (27–120)	65.1 (28–134)	0.32	51.0 (27–103)	65.1 (28–134)	<0.001
Platelet Count×10^9^/L	101.0 (75–130)	100.0 (74–134)	0.73	103.9 (76–136)	100.0 (74–134)	0.67

PS indicates propensity score; n, number of patients; %, number of patients in percent of total per group; M, median; IQR, interquartile range; MI, myocardial infarction; ICU LOS, length of stay on the intensive care unit.

*Composite outcome is any postoperative myocardial infarction, stroke, pneumonia, renal failure with dialysis or cardiac arrest during hospitalization.

After propensity score matching, there were significantly more adverse outcomes in the potentially exposed group (20.9% versus 12.0%, difference = 8.9%; 95% CI 3.6 to 15.1%, P<0.001), and a statistically non-significant difference in patient in-hospital mortality with 5.9% in the potentially contaminated versus 3.5% in the control group (difference = 2.4%; 95% CI −0.4 to 3.0%, P = 0.1). The median ICU length of stay was significantly longer by 14.1 hours (interquartile range −26.6 to 79.8 hours, S = 3299, P = 0.0004). The median lowest platelet counts were similar with 100.0×10^9^/L in the potentially exposed and 100.8×10^9^/L in the control group (interquartile range −30.0 to 33.5, S = 385, P = 0.67).

After adjusting for age, race, number of co-morbidities, left ventricular ejection fraction, cardiopulmonary bypass time, administration of Tranexamic acid, and experience of the surgeon, contaminated heparin proved to be a significant factor for the composite postoperative complication outcome with an odds ratio of 2.0 (95% CI, 1.4 to 3.0, P<0.001) (Table 2). Contaminated heparin did not prove to be a significant predictor for in-hospital mortality (Odds ratio 1.9, 95% CI 0.9 to 3.5, P = 0.11, other odds ratios not shown in detail), but it revealed being significantly associated with a longer stay on the ICU postoperatively (Hazard ratio 1.2, 95% CI 1.0 to 1.4, P = 0.038) (Table 3).

**Table 2:** Logistic Regression Model of Composite Outcome* during Heparin Contamination.

Covariates	Levels	Coefficient (β)	SE	t-Value	OR (95% CI)	P
CH-Exposure		0.70	0.20	3.42	2.0 (1.4–3.0)	<0.001
Age in years	18–44	reference	reference	reference	reference	
	45–74	0.37	0.30	1.22	1.5 (0.8–2.6)	0.22
	≥75	0.88	0.34	2.59	2.4 (1.2–4.7)	<0.01
Race		0.14	0.30	0.48	1.2 (0.6–2.1)	0.63
Comorbidities #	0	reference	reference	reference	reference	
	1–4	0.61	0.49	1.26	1.8 (0.7–4.8)	0.21
	≥5	1.07	0.53	2.00	2.9 (1.0–8.3)	0.045
LVEF	≥55%	reference	reference	reference	reference	
	40–54%	0.55	0.23	2.41	1.7 (1.1–2.7)	0.016
	<40%	0.94	0.23	4.17	2.6 (1.7–4.0)	<0.001
CPB in minutes	0	reference	reference	reference	reference	
	1–179	0.14	0.33	0.44	1.2 (0.6–2.2)	0.66
	≥180	1.04	0.37	2.84	2.8 (1.4–5.9)	0.005
TXA		−0.21	0.20	−1.08	0.8 (0.6–1.2)	0.28
Surgeon		−0.52	0.29	−1.81	0.6 (0.3–1.0)	0.07

SE indicates standard error; OR, odds ratio; CI, confidence interval; CH, contaminated heparin; #, number of comorbidities; LVEF, left ventricular ejection fraction; CPB, cardiopulmonary bypass time; TXA, tranexamic acid. Overall fit: 2 Log likelihood ratio = 65.9, P<0.0001; Wald F = 5.8 on 12 and 906 degrees of freedom, P<0.0001; Hosmer-Lemeshow: X^2^ = 4.9, Degrees of freedom = 8, P = 0.87.

*Composite outcome is any postoperative myocardial infarction, stroke, pneumonia, dialysis or cardiac arrest.

**Table 3:** Proportional Hazards Model of ICU-LOS during Heparin Contamination.

Covariates	Levels	Coefficient (β)	SE	z-Value	HR (95% CI)	P
CH-Exposure		0.17	0.08	2.08	1.2 (1.0–1.4)	0.038
Age in years	18–44	reference	reference	reference	reference	
	45–74	0.35	0.09	3.69	1.4 (1.2–1.7)	<0.001
	≥75	0.66	0.11	5.74	1.9 (1.5–2.4)	<0.0001
Race		0.08	0.10	0.81	1.1 (0.9–1.3)	0.42
Comorbidities #	0	reference	reference	reference	reference	
	1–4	0.17	0.13	1.30	1.2 (0.9–1.5)	0.19
	≥5	0.55	0.15	3.75	1.7 (1.3–2.3)	<0.001
LVEF	≥55%	reference	reference	reference	reference	
	40–54%	0.21	0.08	2.43	1.2 (1.0–1.5)	0.015
	<40%	0.45	0.08	5.62	1.6 (1.3–1.8)	<0.0001
CPB in minutes	0	reference	reference	reference	reference	
	1–179	0.19	0.12	1.63	1.2 (1.0–1.5)	0.10
	≥180	0.59	0.14	4.13	1.8 (1.4–2.4)	<0.0001
TXA		0.11	0.07	1.64	1.1 (1.0–1.3)	0.10
Surgeon		−0.38	0.10	−3.93	0.7 (0.6–0.8)	<0.0001

ICU-LOS indicates length of stay on the intensive care unit; SE, standard error; HR, hazard ratio; CI, confidence interval; CH, contaminated heparin; LVEF, left ventricular ejection fraction; CPB, cardiopulmonary bypass time; TXA, tranexamic acid.

The subgroup analysis for patients being on an angiotensin-converting-enzyme inhibitor or an angiotensin-receptor blocker revealed a potentially protective effect with respect to the composite outcome within the control (9.5% versus 14.5%, difference = 5.0%, 95% CI 0.8 to 9.2%, P = 0.02), but not within the exposed group (Table 4). The largest difference was seen between patients being on an angiotensin-converting-enzyme inhibitor or an angiotensin-receptor blocker receiving regular heparin in comparison to patients receiving contaminated heparin and not taking an angiotensin-converting-enzyme inhibitor or an angiotensin-receptor blocker (9.5% and 23.9% respectively, difference = 14.4%, 95% CI 6.9 to 23.3%, P = 0.013).

**Table 4:** Subgroup Analysis of ACEI and ARB: Regular versus Potentially Contaminated Heparin.

	Events*/Control	Events*/Exposed	P
**On ACEI/ARB**	38/402 (9.5%)	18/103 (17.5%)	0.021
**No ACEI/ARB**	75/516 (14.5%)	28/117 (23.9%)	0.013
**P**	0.02	0.24	

ACEI indicates angiotensin-converting-enzyme inhibitor; ARB, angiotensin-receptor blocker.

*Events of composite outcome.

### Summary of findings from independently repeated statistical analysis

In general the findings of the independently repeated statistical analysis were similar to those found initially. Most notably, for the composite postoperative complication outcome using logistic regression, potential exposure to contaminated heparin had a statistically significant effect with p value = 0.0025 and odds ratio = 1.897. This analysis was repeated for other matched samples, and the p value was persistently below 0.05. For mortality, potential exposure to contaminated heparin showed a statistically insignificant effect with p value = 0.0772 and odds ratio = 1.908. In contrast to the initial analysis, potential exposure to contaminated heparin was associated with a non-significant (P = 0.065) risk for increased intensive care unit stay.

## Discussion

This single center study found that OSCS heparin contamination might have been associated with increased morbidity and length of ICU stay following cardiac surgery. This is the first study to show that OSCS contamination might have had a broad negative impact rather than sporadically and often transiently causing adverse events related to anaphylactoid reactions. However, contrary to previous suggestions, this study found no evidence that OSCS heparin worsened thrombocytopenia, or by implication might have promoted heparin-induced thrombocytopenia. This trial also did not find that OSCS heparin, through bradykinin production, improved outcomes following cardiac surgery.

The credibility of the main findings is supported by several factors. First, the results were similar with and without propensity score matching. Second, the estimated incidence for each of the individual complications in the composite outcome was higher in the patients who potentially received OSCS contaminated heparin. Third, the results are mechanistically plausible, as hypotension-induced organ dysfunction and bradykinin-triggered inflammation with vascular permeability seen with angioedema might have driven the adverse outcomes [20]. If OSCS contaminated heparin was associated with adverse outcomes, mechanisms other than bradykinin production might have been involved as patients in the control group who were taking an angiotensin-converting-enzyme inhibitor (also associated with increased bradykinin [11]) had fewer complications. The protective effect of angiotensin-converting-enzyme inhibitors could be attributable to blocking the increased expression of pro-inflammatory factors triggered by angiotensin II and by reducing the stimulation of superoxide formation [21], [22].

This epidemiological study provides indirect evidence that anaphylactoid reactions are probably associated with worse outcomes, even if the acute episode is managed appropriately. This inference is based on four key assumptions. The first is that OSCS heparin was actually associated with increased morbidity and the results in this study were not just spurious. The second is that the anaphylactoid reactions to OSCS heparin were managed appropriately when they occurred in the operating room. The third assumption is that the adverse outcomes were attributable to anaphylactoid reactions rather than to some other consequence of OSCS. Finally, it is assumed that patients included in this study actually received OSCS contaminated heparin.

This study had important limitations. As this was a non-randomized, retrospective, observational study, there might have been uncontrollable, unknown or hidden confounding factors [17], [23]. We attempted to address this through propensity score matching followed by multivariable regression analysis. However, important data known to impact outcomes after cardiac surgery, such as blood component transfusions [24], were incomplete, and could not be included in the analyses. This was a single center study with relatively low numbers; as such the findings are potentially fragile and imprecise. Finally, it is unknown which specific patients received contaminated heparin and detailed hemodynamic data from the operating room were not available.

In conclusion, we compared the outcomes of patients undergoing adult cardiac surgery who potentially received OSCS contaminated heparin and those who received uncontaminated heparin. Exposure to contaminated heparin appears to have been associated with increased risk of postoperative complications and longer ICU stay. This result must be regarded only as hypothesis-generating as it was an observational study from a single center in the United States. In order to clarify whether or not there was an increase in morbidity and perhaps mortality for patients undergoing heart surgery during the period that OSCS heparin was available, we have approached the Society for Thoracic Surgeons national organization and have requested access to national STS data so that we can repeat our analysis on a much larger scale, which should provide sufficient patient numbers and a broadly representative cohort in order to answer the hypothesis with confidence and precision.

## Supporting Information

Table S1 **Characteristics of all Covariates Before and After Propensity Score Matching during Heparin Contamination.** PS indicates propensity score; n, number of patients; %, number of patients in percent of total per group; m, mean; SD, standard deviation; d, standardized mean difference; BMI, body mass index; ACEI, angiotensin-converting-enzyme inhibitor; ARB, angiotensin-receptor blocker; CVD, cerebrovascular disease; PAH, pulmonary hypertension; NIDDM, non–insulin-dependent diabetes mellitus; IDDM, insulin-dependent diabetes mellitus; MI, myocardial infarction; CAD, coronary artery disease; LVEF, left ventricular ejection fraction; IABP, intra-aortic balloon pump; CABG, coronary artery bypass graft. *Valvular insufficiency or stenosis of moderate or severe Grade. †Surgery performed by an Instructor or Assistant Professor in Cardiac Surgery. **‡**Previous Cardiovascular Intervention, either Surgical or Non-surgical. §Any cardiac operation involving a heart valve with or without a concomitant coronary artery bypass graft.
(DOCX)
