# Supplementary material for: Contaminated Heparin and Outcomes after Cardiac Surgery: A Retrospective Propensity-Matched Cohort Study
Source: PLoS One. 2014 Aug 27;9(8):e106096. doi: 10.1371/journal.pone.0106096 (PMC4146562; doi:10.1371/journal.pone.0106096)
Supplement: Table S1 — Characteristics of all Covariates Before and After Propensity Score Matching during Heparin Contamination. PS indicates propensity score; n, number of patients; %, number of patients in percent of total per group; m, mean; SD, standard deviation; d, standardized mean difference; BMI, body mass index; ACEI, angiotensin-converting-enzyme inhibitor; ARB, angiotensin-receptor blocker; CVD, cerebrovascular disease; PAH, pulmonary hypertension; NIDDM, non–insulin-dependent diabetes mellitus; IDDM, insulin-dependent diabetes mellitus; MI, myocardial infarction; CAD, coronary artery disease; LVEF, left ventricular ejection fraction; IABP, intra-aortic balloon pump; CABG, coronary artery bypass graft. *Valvular insufficiency or stenosis of moderate or severe Grade. †Surgery performed by an Instructor or Assistant Professor in Cardiac Surgery. ‡Previous Cardiovascular Intervention, either Surgical or Non-surgical. §Any cardiac operation involving a heart valve with or without a concomitant coronary artery bypass graft. (DOCX) [file pone.0106096.s001.docx]

|  | **Before PS Matching** | | | | **After PS Matching** | | | | |
| --- | --- | --- | --- | --- | --- | --- | --- | --- | --- |
|  | **Control** | **Exposed** |  |  | **Control** | **Exposed** |  |  |  |
|  | **(n=1197)** | **(n=220)** |  |  | **(n=918)** | **(n=220)** |  |  |  |
| **Covariate** | **m(SD) or n(%)** | **m(SD) or n(%)** | **d** | **P** | **m(SD) or n(%)** | **m(SD) or n(%)** | **d** | **Variance Ratio** | **P** |
| PS | 0.15 (0.07) | 0.19 (0.07) | 0.54 | <0.001 | 0.19 (0.07) | 0.19 (0.07) | 0.00 | 0.99 | 0.56 |
| Age in years | 60.5 (14.9) | 60.7 (14.8) | -0.01 | 0.85 | 59.9 (14.9) | 60.7 (14.8) | 0.00 | 1.05 | 0.48 |
| Gender (male) | 790 (66.0) | 138 (62.7) | 0.07 | 0.35 | 573 (62.4) | 138 (62.7) | 0.01 | 0.99 | 0.93 |
| Race (white) | 1028 (85.9) | 195 (88.6) | -0.09 | 0.27 | 816 (88.9) | 195 (88.6) | 0.01 | 0.98 | 0.91 |
| BMI in kg/m^2^ | 28.9 (6.1) | 29.1 (6.9) | 0.07 | 0.59 | 29.3 (6.4) | 29.1 (6.9) | 0.01 | 1.01 | 0.84 |
| ACEI or ARB | 516 (43.1) | 103 (46.8) | 0.07 | 0.31 | 419 (45.6) | 103 (46.8) | 0.02 | 0.99 | 0.75 |
| CVD | 204 (17.0) | 40 (18.2) | 0.03 | 0.68 | 168 (18.3) | 40 (18.2) | 0.01 | 0.98 | 0.97 |
| Chronic Lung Disease | 85 (7.1) | 16 (7.3) | 0.01 | 0.93 | 63 (6.9) | 16 (7.3) | 0.01 | 0.98 | 0.83 |
| PAH | 435 (36.3) | 53 (24.1) | -0.29 | <0.001 | 221 (24.1) | 53 (24.1) | 0.00 | 1.00 | 1.00 |
| Renal Failure | 54 (4.5) | 12 (5.5) | 0.04 | 0.54 | 47 (5.1) | 12 (5.5) | 0.02 | 0.90 | 0.84 |
| NIDDM | 200 (16.7) | 30 (13.6) |  |  | 135 (14.7) | 30 (13.6) |  |  |  |
| IDDM | 138 (11.5) | 16 (7.3) | -0.20 | 0.06 | 66 (7.2) | 16 (7.3) | 0.02 | 0.95 | 0.92 |
| Stable Angina | 245 (20.5) | 39 (17.7) |  |  | 189 (20.6) | 39 (17.7) |  |  |  |
| Unstable Angina | 143 (11.9) | 19 (8.6) | -0.15 | 0.18 | 72 (7.8) | 19 (8.6) | -0.01 | 1.00 | 0.62 |
| MI >21 days ago | 224 (18.7) | 35 (15.9) |  |  | 172 (18.7) | 35 (15.9) |  |  |  |
| MI ≤21 days ago | 177 (14.8) | 31 (14.1) | -0.06 | 0.55 | 118 (12.9) | 31 (14.1) | -0.01 | 0.95 | 0.59 |
| CAD: 1 or 2 vessel | 267 (22.3) | 48 (21.8) |  |  | 199 (21.7) | 48 (21.8) |  |  |  |
| CAD: 3 vessel | 352 (29.4) | 67 (30.5) | 0.02 | 0.95 | 285 (31.0) | 67 (30.5) | 0.02 | 0.97 | 0.99 |
| Valvular Insufficiency*: 1 | 368 (30.7) | 65 (29.5) |  |  | 256 (27.9) | 65 (29.5) |  |  |  |
| Valvular Insufficiency*: ≥2 | 146 (12.2) | 15 (6.8) | -0.19 | <0.05 | 65 (7.1) | 15 (6.8) | -0.02 | 1.09 | 0.89 |
| Valvular Stenosis* | 254 (21.2) | 34 (15.5) | -0.16 | 0.05 | 135 (14.7) | 34 (15.5) | -0.01 | 1.01 | 1.00 |
| Endocarditis | 66 (5.5) | 9 (4.1) | -0.07 | 0.39 | 35 (3.8) | 9 (4.1) | 0.03 | 0.88 | 0.85 |
| LVEF | 49.2 (15.5) | 48.6 (16.4) | 0.03 | 0.59 | 49.1 (15.3) | 48.6 (16.4) | 0.01 | 0.94 | 0.66 |
| Cardiogenic Shock | 94 (7.9) | 13 (5.9) | -0.08 | 0.32 | 58 (6.3) | 13 (5.9) | 0.00 | 0.98 | 0.82 |
| Junior Faculty Surgery† | 236 (19.7) | 38 (17.3) | -0.06 | 0.40 | 170 (18.5) | 38 (17.3) | 0.00 | 1.00 | 0.67 |
| Urgent/Emergent Operation | 148 (12.4) | 21 (9.5) | -0.10 | 0.24 | 90 (9.8) | 21 (9.5) | 0.01 | 0.97 | 0.91 |
| Re-Operation | 239 (20.0) | 44 (20.0) | 0.00 | 0.99 | 181 (19.7) | 44 (20.0) | -0.01 | 1.01 | 0.92 |
| Previous Cardiac Intervention‡ | 369 (30.8) | 83 (37.7) | 0.14 | <0.05 | 345 (37.5) | 83 (37.7) | 0.00 | 1.00 | 0.97 |
| IABP | 53 (4.4) | 9 (4.1) | -0.02 | 0.82 | 37 (4.0) | 9 (4.1) | 0.00 | 1.00 | 0.97 |
| Operation: CABG | 403 (33.7) | 88 (40.0) |  |  | 362 (39.4) | 88 (40.0) |  |  |  |
| Operation: Valve§ | 549 (45.9) | 86 (39.1) |  |  | 359 (39.1) | 86 (39.1) |  |  |  |
| Operation: Other | 245 (20.1) | 46 (20.9) | -0.08 | 0.13 | 197 (21.5) | 46 (20.9) | -0.02 | 0.99 | 0.98 |
